# Prenatal diffusion-weighted MRI in growth-restricted fetuses: early diffusion changes beyond Doppler findings: a retrospective cohort study

**DOI:** 10.1186/s12884-026-09164-4

**Published:** 2026-04-25

**Authors:** Bircan Yildirim Baydemir, Sahin Kaan Baydemir, Suat Fitoz, Acar Koc

**Affiliations:** 1 School of Medicine, Department of Obstetrics and Gynecology, Ankara University, Ankara, Türkiye; 2 Department of Obstetrics and Gynecology, Gynecologic Oncology, Etlik City Hospital, Ankara, Türkiye; 3 School of Medicine, Department of Radiology, Pediatric Radiology, Ankara University, Ankara, Türkiye

**Keywords:** Fetal growth restriction, Small for gestational age, Diffusion-weighted MRI, Apparent diffusion coefficient, Fetal brain imaging, Cerebroplacental ratio

## Abstract

**Background:**

Although fetal growth restriction (FGR) and small for gestational age (SGA) both denote impaired fetal growth, they are distinct clinical entities defined by different diagnostic criteria. FGR is primarily associated with placental insufficiency and chronic hypoxia, which may result in progressive cerebral injury even before overt clinical manifestations become apparent. However, direct comparisons of brain diffusion characteristics between FGR and SGA fetuses remain limited. To evaluate fetal brain diffusion changes in growth-restricted (FGR) and small-for-gestational-age (SGA) fetuses using diffusion-weighted magnetic resonance imaging (DW-MRI), and to correlate findings with Doppler ultrasound parameters.

**Methods:**

In this retrospective study, we included singleton pregnancies diagnosed with FGR or SGA who underwent fetal brain MRI after 30 weeks of gestation. FGR was defined based on estimated fetal weight or abdominal circumference <10th percentile with abnormal Doppler indices, while SGA fetuses had similar biometric criteria but normal Dopplers. Apparent diffusion coefficient (ADC) values were measured in eleven brain regions including frontal and occipital white matter, thalami, centrum semiovale, pons, and cerebellar hemispheres and compared between FGR and SGA groups. Doppler indices and perinatal outcomes were also analyzed.

**Results:**

A total of 44 patients (30 FGR, 14 SGA) were included. FGR fetuses had significantly higher umbilical artery pulsatility index, smaller biparietal and transverse cerebellar diameters, and lower ADC values in frontal white matter compared to SGA fetuses. ADC values in other brain regions were not significantly different. Lower 1-minute Apgar scores, lower cord pH values, and higher NICU admission rates were more frequent in the FGR group.

**Conclusion:**

DW-MRI may identify early brain alterations in FGR fetuses before overt clinical signs. Combined with Doppler findings, MRI can enhance risk stratification and guide timing of delivery.

## Background

Fetal growth restriction (FGR) and small for gestational age (SGA) are both defined as an estimated fetal weight (EFW) below the 10th percentile for gestational age [1, 2]. While SGA fetuses typically carry a lower risk of complications, FGR represents a more severe condition and is a leading cause of perinatal morbidity and mortality [3]. Newborns with FGR are more likely to experience birth acidosis, low Apgar scores, and require neonatal intensive care unit (NICU) admission [4]. Although both conditions denote impaired fetal growth, their underlying pathophysiological mechanisms differ substantially. FGR is primarily associated with placental insufficiency and confers an increased risk of fetal hypoxia and subsequent cerebral injury. Nevertheless, studies directly comparing brain diffusion alterations between FGR and SGA fetuses remain limited.

FGR is classified as early-onset (before 32 weeks) or late-onset (≥ 32 weeks) based on gestational age at diagnosis [5]. An EFW below the 3rd percentile combined with abnormal umbilical artery (UA) Doppler findings indicates a more severe form, associated with increased neonatal morbidity and mortality [6, 7].

Intrauterine hypoxia in FGR can lead to fetal brain injury due to limited compensatory capacity. Brain-sparing responses, including peripheral vasoconstriction and cerebral vasodilation mediated by adenosine and nitric oxide, aim to improve cerebral oxygenation. With ongoing hypoxia, perfusion is redirected to vital areas such as the basal ganglia and pons [8, 9]. Absent or reversed UA end-diastolic flow indicates high placental resistance, while increased cerebral diastolic flow suggests compensatory vasodilation [8, 10]. This redistribution is assessed via middle cerebral artery (MCA)Doppler and the cerebroplacental ratio (CPR) [11] a key delivery timing criterion per International Society of Ultrasound in Obstetrics and Gynecology (ISUOG) [1]. CPR is calculated as MCA PI divided by UA PI and predicts adverse outcomes with 66% sensitivity and 85% specificity [9, 12, 13]. However, the threshold for irreversible brain injury remains uncertain.

Diffusion-weighted MRI (DW-MRI), increasingly applied in obstetric imaging, enables assessment of microstructural brain changes by measuring water diffusion, quantified as the apparent diffusion coefficient (ADC). Hypoxia-related extracellular edema alters ADC values, particularly in susceptible brain regions. Previous studies, though limited, have reported lower ADC values in FGR fetuses compared to normally grown fetuses, indicating early hypoxic injury [8, 14].

In this study, we compared FGR fetuses with abnormal UA Doppler findings to SGA fetuses with normal Doppler results. To our knowledge, such a direct comparison using fetal brain DW-MRI has not been well characterized in the literature. Our aim was to evaluate the utility of fetal brain MRI in detecting early hypoxic brain changes and to explore its potential role in guiding delivery timing.

## Methods

This retrospective study included patients who were referred to the perinatology clinic of the Department of Obstetrics and Gynecology, Ankara University School of Medicine, Ankara, Türkiye between June 2017 and June 2018. Ethical approval was obtained from the University Ethics Committee’s Institutional Review Board (approval no: 11-743-18). Singleton pregnancies diagnosed with either FGR or SGA and evaluated by fetal MRI were eligible for inclusion. Additional inclusion criteria were a gestational age of ≥ 30 weeks at the time of diagnosis and evaluation at our institution, including Doppler assessment and fetal MRI. Exclusion criteria were: multiple pregnancies, known chromosomal abnormalities, congenital anomalies, confirmed or suspected intrauterine infection, and refusal to undergo MRI. Gestational age was determined by first-trimester crown–rump length measurement.

Patients were divided into two groups based on ISUOG criteria [1, 15]: the FGR group included fetuses with an abdominal circumference (AC) or estimated fetal weight (EFW) below the 3rd percentile, or absent end-diastolic flow in the UA, or AC/EFW below the 10th percentile combined with uterine artery pulsatility index (PI) >95th percentile and/or UA-PI > 95th percentile. The SGA group included fetuses with AC/EFW < 10th percentile but with normal Doppler findings. MRI examinations were performed on the day of diagnosis or within 24 h thereafter; therefore, gestational age at MRI closely reflected gestational age at diagnosis and was used for all subsequent analyses. All conventional MRI sequences showed normal fetal brain morphology without structural anomalies. Patients in both groups were followed until delivery in accordance with current clinical guidelines [16, 17]. Antenatal and perinatal data were collected for all cases, except for two patients who delivered at another facility.

### MRI protocol

All fetal brain MRIs were performed using a standardized protocol on a 1.5 Tesla MR scanner (Philips, Insignia) with a conventional phased-array body coil. The following conventional sequences were used: a T2-weighted single-shot turbo spin-echo (T2-TSE) sequence and a steady state acquisition sequence (B-FFE) with a 4 mm slice thickness without gap, which were obtained in three orthogonal planes. T1-weighted spin-echo (T1-SE) with a 4 mm slice thickness was also obtained in the axial plane. All MRI examinations were first reviewed for image quality. Scans affected by severe motion artifacts caused by maternal breathing or fetal movements were excluded from the analysis (*n* = 3; 2 in the FGR group and 1 in the SGA group).

**Fig. 1:**
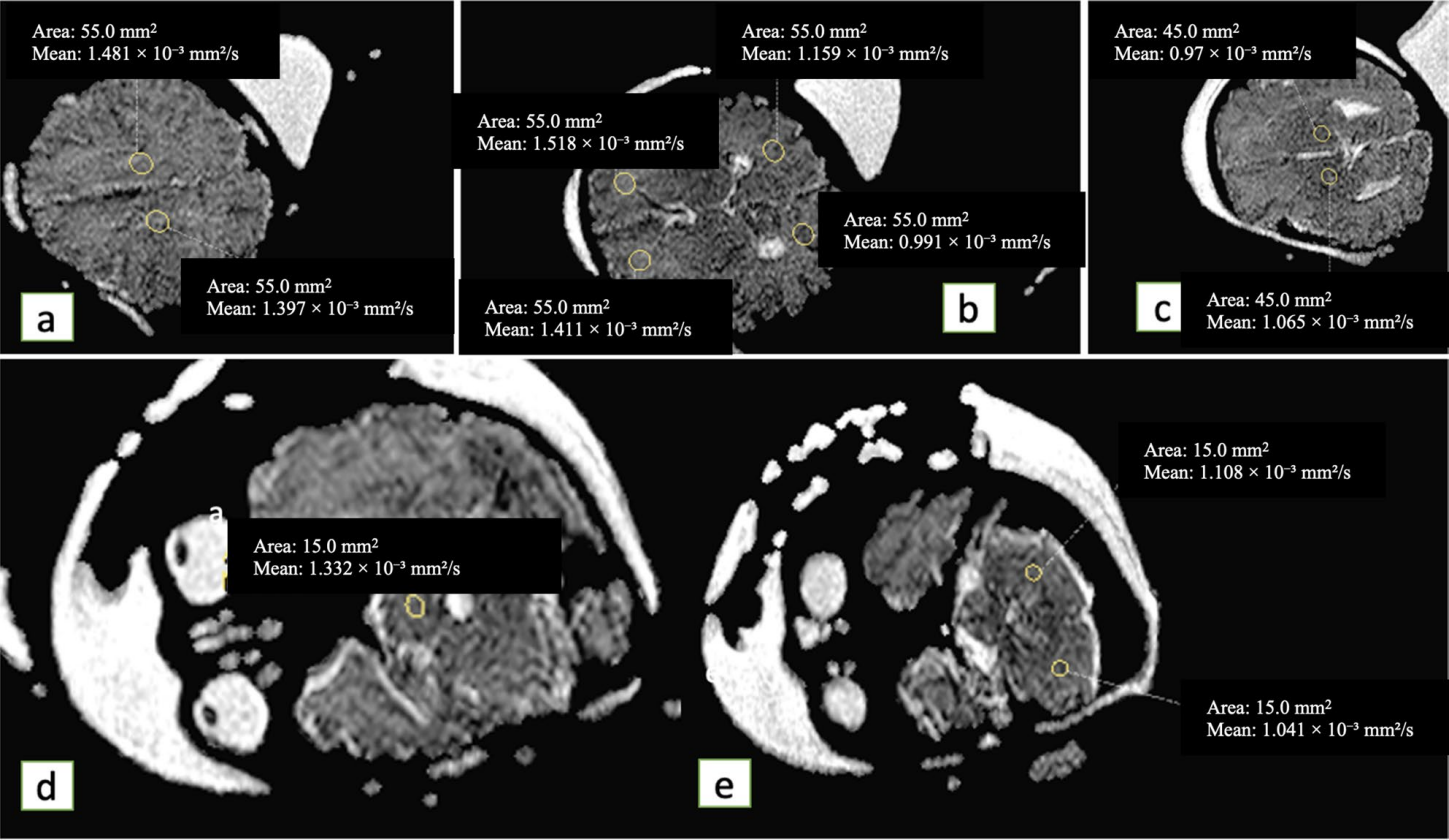
Representative axial ADC maps showing region-of-interest (ROI) placement in eleven different brain regions: **a** centrum semiovale, **b** frontal and occipital white matter, **c** bilateral thalami, **d** pons, and **e** cerebellar hemispheres

DWI was performed using single-shot spin-echo-planar imaging in the axial plane with the following parameters: 24 images in six non-collinear axis directions; EPI factor, 109; FOV, 240 mm; matrix, 240 × 165; slice thickness, 4 mm with a 1-mm gap. Diffusion-gradient values were b = 0 and b = 1000 s/mm^2^. ADC maps were generated automatically on the main MRI console. ADC values were obtained by placing regions of interest (ROI) in eleven different brain regions on axial ADC maps, using consistent neuroanatomical landmarks and similar ROI sizes including the frontal and occipital white matter, thalami, centrum semiovale, pons, and cerebellar hemispheres (Fig. 1). ADC values of frontal and occipital white matter were measured at the level of the frontal horn and the atria on the same axial slice, with an average ROI surface of 30–60 mm2 for each hemisphere. On the first axial slice located above the ventricles, ROI with an average surface of 30–60 mm2 was placed in the center of the white matter of the centrum semiovale for each hemisphere, and at the level of the basal ganglia, ADC values of the bilateral thalamus with an average ROI surface of 30–60 mm2 were calculated. With an avarege ROI surface of 5–15 mm2, at the level of the middle cerebellar peduncles, ADC values of both cerebellar white matter and one measurement for the pons at the level of the central pons were made. For each ROI, a mean ± SD ADC value (×10 − 3 mm^2^/s) was obtained. Brain biometry measurements were performed on transverse T2-weighted images on axial ADC maps, including biparietal diameter, fronto–occipital diameter, and transverse cerebellar diameter. Biparietal diameter was measured as the maximum transverse distance between the parietal bones from the outer to the inner calvarial surface, fronto–occipital diameter as the maximal distance between the frontal and occipital bones, and transverse cerebellar diameter as the widest transverse measurement of the cerebellum. All ADC and brain biometry measurements were performed by a single pediatric radiologist with 19 years of experience in fetal MRI, who was blinded to all clinical information. All measurements were repeated in a separate session, and the mean values were used for analysis.

### Statistical analysis

Statistical analysis was performed using the software IBM SPSS 11.5 (SPSS Inc., Chicago, IL, USA). Kolmogorov-Smirnov/Shapiro-Wilk tests were used to determine distribution of variables. Descriptive analyses were presented as means and standard deviations for normally distributed variables or medians and range for non-normally distributed variables. Statistical analyses were performed using independent-samples Student’s t-test for normally distributed continuous data and Mann–Whitney U test for non-normally distributed continuous data. The Chi-square test or Fisher’s exact test, where appropriate, were used to compare the proportions in different groups. ADC measurements that showed significant between-group differences in unadjusted analyses were further adjusted for gestational age at MRI using analysis of covariance (ANCOVA). A p-value < 0.05 was considered statistically significant. Given the retrospective design and fixed sample size, a sensitivity power analysis was performed. Assuming a two-sided α level of 0.05 and 80% statistical power, the minimum detectable standardized difference for the primary outcome (frontal white matter ADC) between the FGR and SGA groups was Cohen’s d = 0.93, corresponding to an absolute difference of approximately 0.22 × 10⁻³ mm²/s based on the pooled standard deviation.

## Results

A total of 70 patients diagnosed with FGR or SGA were followed at our perinatology clinic. Of these, 30 patients with FGR and 14 with SGA met the inclusion criteria and were enrolled in the study. Maternal demographic characteristics are presented in Table 1. There were no statistically significant differences between the groups in terms of maternal age, gravida, parity, systemic diseases, adverse obstetric history, or gestational age at the time of MRI. The mean gestational age at the time of MRI was 33.3 ± 3.1 weeks in the FGR group and 34.0 ± 2.3 weeks in the SGA group. The FGR group showed higher UA pulsatility index (PI) values than the SGA group (1.23 ± 0.47 vs. 0.98 ± 0.11; *p* = .002), whereas MCA PI and CPR were similar between the two groups.

**Table 1:** Baseline maternal and fetal doppler characteristics of the study population

	FGR (*n* = 30)	SGA (*n* = 14)	*p* value
Maternal Characteristics			
Age, years, mean ± SD	26.8 ± 5.1	24.2 ± 3.7	0.128
Gravida, median (IQR)	1 (1–2)	2 (1–2)	0.899
Parity, median (IQR)	0 (0–1)	1 (0–1)	0.942
Systemic diseases, n (%)	17 (56.7)	6 (42.9)	0.393
History of Adverse Perinatal Outcome n (%)	7 (23.3)	2 (14.3)	0.695
Gestational age at MRI, weeks, mean ± SD	33.3 ± 3.1	34.0 ± 2.3	0.062
Fetal Findings at Doppler			
Umblical Artery Doppler PI, mean ± SD	1.23 ± 0.47	0.98 ± 0.11	**0.002**
Middle Cerebral Artery Doppler PI, mean ± SD	1.51 ± 0.47	1.56 ± 0.34	0.531
Cerebroplacental Ratio (CPR), mean ± SD	1.37 ± 0.62	1.53 ± 0.32	0.054

Bold values indicate statistical significance (*p* < 0.05)

*SD* Standard deviation

*T test or Chi-square test

**Fig. 2:**
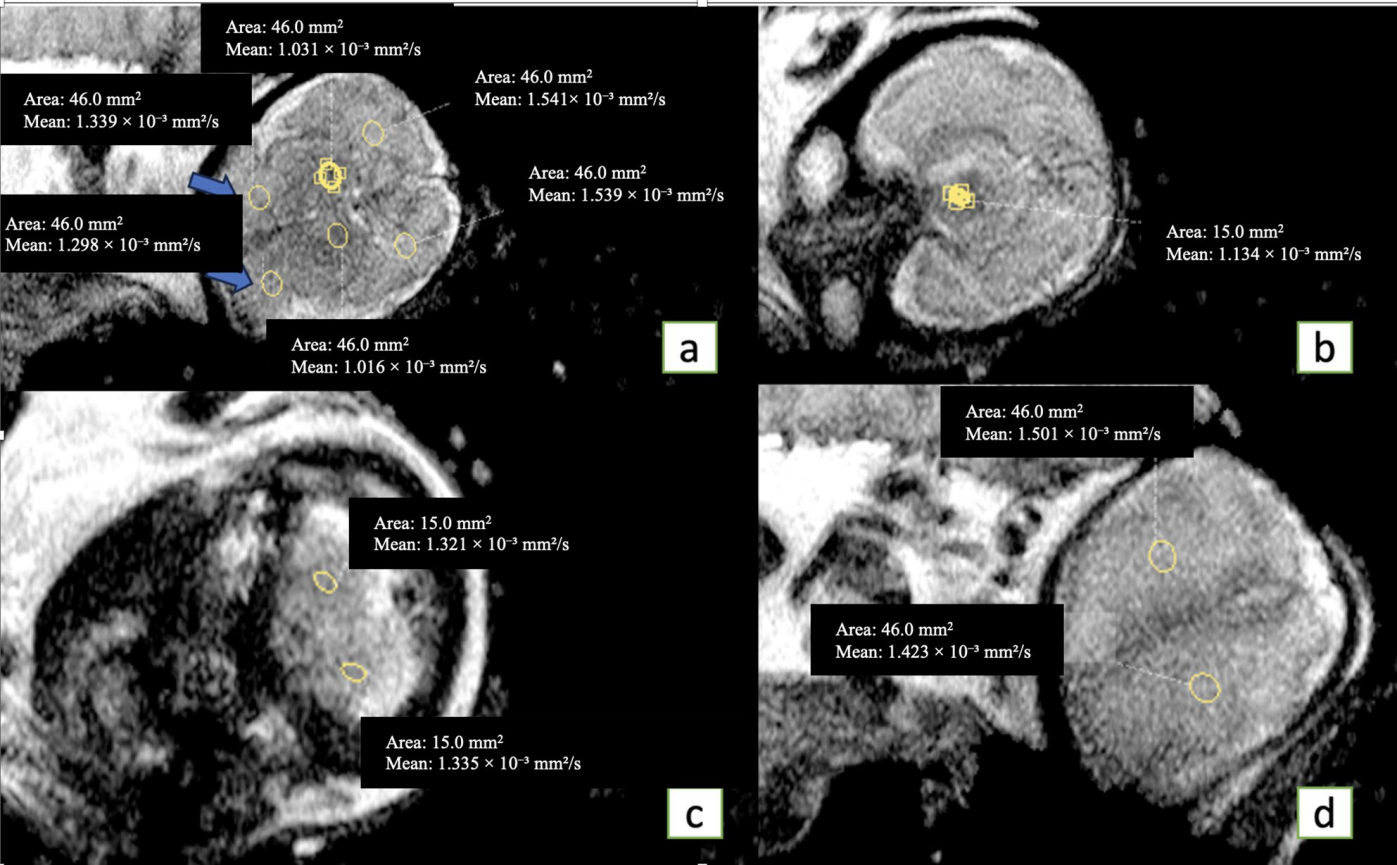
Representative ADC measurements from different brain regions in an FGR fetus with reduced ADC values in the frontal white matter. **A** ROI placement in the frontal white matter (left, arrows), bilateral thalami (center), and occipital white matter (right) **b** Pons **c** Cerebellum **d** Centrum semiovale

Fetal brain biometry and regional ADC measurements in the FGR and SGA groups are summarized in Table 2. Both biparietal and transverse cerebellar diameters were significantly smaller in the FGR group compared to the SGA group (80.3 ± 9.4 vs. 85.8 ± 2.9, *p* = .002; and 41.0 ± 7.2 vs. 44.7 ± 4.0, *p* = .002, respectively). Additionally, ADC values of the frontal white matter were significantly lower in the FGR group than in the SGA group (*p* = .003 for the right frontal lobe and *p* = .026 for the left frontal lobe). Representative examples of ADC measurements from different brain regions in an FGR fetus are shown in Fig. 2, with reduced ADC values particularly evident in the frontal white matter. After adjustment for gestational age at MRI, frontal white matter ADC values remained significantly higher in the SGA group compared with the FGR group (right: adjusted means 1.759 vs. 1.632, *p*=.005; left: 1.768 vs. 1.638, *p*=.008). No significant differences were observed in the ADC values of other brain regions, including the occipital white matter, centrum semiovale, thalami, cerebellum, and pons.

**Table 2:** Fetal brain biometry and ADC measurements in FGR and SGA groups

	FGR (*n* = 30)	SGA (*n* = 14)	*p* value
Fetal Findings at MRI – Diameters (mm)			
Biparietal Diameter (BPD), mean ± SD	80.3 ± 9.4 (76.8–83.8)	85.8 ± 2.9 (84.1–87.5)	**0.002**
Fronto-Occipital Diameter (FOD), mean ± SD	97.6 ± 10.4 (93.7–101.5)	101.3 ± 5.5 (98.1–104.5)	0.298
Transverse Cerebellar Diameter (TCD), mean ± SD	41.0 ± 7.2 (38.3–43.7)	44.7 ± 4.0 (42.4–47.0)	**0.002**
Fetal Findings at MRI – ADC measurements (10^-3^mm^2^/s )			
Frontal WM – right, mean ± SD	1.63 ± 0.25 (1.54–1.72)	1.71 ± 0.19 (1.60–1.82)	**0.003**
Frontal WM – left, mean ± SD	1.66 ± 0.22 (1.53–1.79)	1.70 ± 0.20 (1.63–1.77	**0.026**
Occipital WM – right, mean ± SD	1.54 ± 0.23 (1.45–1.63)	1.61 ± 0.14 (1.53–1.69)	0.292
Occipital WM – left, mean ± SD	1.60 ± 0.23 (1.51–1.69)	1.56 ± 0.16 (1.47–1.65)	0.641
Centrum Semiovale – right, mean ± SD	1.55 ± 0.17 (1.49–1.61)	1.57 ± 0.21 (1.45–1.69)	0.722
Centrum Semiovale – left, mean ± SD	1.51 ± 0.17 (1.45–1.57)	1.55 ± 0.17 (1.45–1.65)	0.585
Thalami – right, mean ± SD	0.88 ± 0.40 (0.73–1.03)	0.97 ± 0.29 (0.80–1.14)	0.460
Thalami – left, mean ± SD	1.05 ± 0.17 (0.99–1.11)	1.06 ± 0.10 (1.00–1.12)	0.566
Cerebellum – right, mean ± SD	1.30 ± 0.15 (1.24–1.36)	1.26 ± 0.10 (1.20–1.32)	0.373
Cerebellum – left, mean ± SD	1.27 ± 0.20 (1.20–1.34)	1.22 ± 0.10 (1.16–1.28)	0.256
Pons, mean ± SD	0.85 ± 0.34 (0.72–0.98)	0.84 ± 0.27 (0.68–1.00)	0.658

Bold values indicate statistical significance (*p* < 0.05)

Values are presented as mean ± SD (95% confidence interval)

*SD* Standard deviation, *WM* White Matter

*T test or Chi-square test

Perinatal outcomes are shown in Table 3. The rate of cesarean delivery was 68.9% in the FGR group and 38.4% in the SGA group. The mean gestational age at birth was 36.14 ± 2.94 weeks in the FGR group and 37.85 ± 0.98 weeks in the SGA group. However, there were no statistically significant differences between the groups in terms of gestational age at delivery or mode of delivery. The interval between fetal MRI and delivery was significantly shorter in the FGR group than in the SGA group (median [IQR]: 7 [11.5] vs. 17.5 [15.8] days, respectively; *p* = .012). The median (IQR) 1-minute Apgar score was significantly lower in the FGR group than in the SGA group [7 ([6–7]) vs. 7 ([7–8]), *p* = .004], whereas 5-minute Apgar scores were comparable between groups. UA pH levels were significantly lower in the FGR group than in the SGA group (7.09 ± 0.11 vs. 7.29 ± 0.09; *p* = .001). Two neonates born at external centers were lost to follow-up. Among the remaining newborns, 16 (38%) required admission to the NICU. NICU admission was significantly more common in the FGR group than in the SGA group (48.2% vs. 15.3%; *p* = .042).

**Table 3:** Perinatal outcomes of the study groups

	FGR (*n* = 29)	SGA (*n* = 13)	*P* value
Caesarean Section, *n* (%)	20 (68.9)	5 (38.4)	0.063
Gestational age at birth, weeks, mean ± SD	36.14 ± 2.94 (35.02–37.26)	37.85 ± 0.98 (37.26–38.44)	0.067
Birth weight, grams, mean ± SD	2003.5 ± 508.2 (1810–2197)	2566.4 ± 316.9 (2375–2758)	0.066
1-minute APGAR scores, median (IQR)	7 (6–7)	7 (7–8)	**0.004**
5-minute APGAR scores, median (IQR)	9 (8.5–9)	9 (9–9)	0.090
Cord blood arterial pH, mean ± SD	7.09 ± 0.11 (7.051–7.137)	7.29 ± 0.09 (7.236–7.344)	**0.001**
Hospitalization at NICU, n (%)	14 (48.2)	2 (15.3)	**0.042**

Bold values indicate statistical significance (*p* < 0.05)

Values are presented as mean ± SD (95% confidence interval) or median (interquartile range), as appropriate

*SD* Standard deviation, *NICU* Neonatal Intensive Care Unit

*T test or Chi-square test

## Discussion

This study evaluated MRI and Doppler findings in fetuses with FGR and SGA. FGR fetuses had smaller biparietal and transverse cerebellar diameters and lower ADC values in the frontal white matter. Although UA Doppler PI was higher in the FGR group, this finding largely reflects the predefined diagnostic criteria and should not be interpreted as an independent result. Of note, MCA PI and CPR were comparable between the two groups, suggesting that overt cerebral redistribution or brain-sparing was not evident in this cohort at the time of assessment. What makes this finding particularly interesting is that frontal white matter ADC values were already reduced in the FGR group despite the absence of Doppler-detectable brain-sparing — raising the possibility that diffusion changes in the fetal brain may emerge before the classic hemodynamic redistribution becomes apparent. The main clinical implications of our study relate to MRI-derived parameters, particularly frontal white matter ADC values. Since brain sparing initially preserves frontal regions before redirecting flow to deeper structures like the basal ganglia and pons, early changes in frontal ADC values may indicate evolving hypoxic injury [8, 18]. These results support the potential of MRI for earlier detection of brain damage and more informed delivery planning.

Previous studies have shown reduced brain volumes and dimensions in FGR fetuses. Peretz et al. and Polat et al. reported that overall brain structures were significantly smaller in FGR cases, likely due to brain sparing mechanisms [19, 20]. Similarly, a prospective cohort study using MRI found that FGR fetuses had reduced occipitofrontal and biparietal diameters compared to normally grown fetuses [21]. Our findings are consistent, showing significantly smaller biparietal and transverse cerebellar diameters in FGR compared to SGA fetuses. 

Diffusion-weighted MRI studies have shown reduced ADC values in FGR fetuses, indicating early ischemic injury. Abdel Razek et al. found globally decreased ADC values in FGR brains [14]. Arthurs et al. demonstrated significantly lower ADC values in specific regions such as the frontal white matter, thalami, centrum semiovale, and pons in severe FGR [8]. In our study, similar regional ADC patterns were observed; however, a statistically significant difference was detected only in the frontal white matter, while no significant differences were found in the other examined regions, possibly due to the inclusion of SGA fetuses—who may also experience mild hypoxia—as controls. Kutuk et al. showed significantly reduced ADC values in periatrial and frontal white matter, thalami, and basal ganglia in FGR fetuses, particularly in those with reversed umbilical artery flow [22]. Similarly, in our cohort, lower frontal white matter ADC values were observed in fetuses with higher UA PI, suggesting a possible relationship between Doppler abnormalities and microstructural brain changes.

A multicenter study reported that SGA fetuses with adverse outcomes had lower frontal white matter ADC values, and proposed a threshold of 1.7 × 10⁻³ mm²/s for risk prediction, though without statistical significance [23]. In line with these findings, lower ADC values were more commonly observed among fetuses with adverse perinatal outcomes, including lower 1-minute Apgar scores, lower UA pH, and higher NICU admission rates. A previous study showed significantly lower ADC values in the cerebellar hemispheres, thalami, and caudate nucleus of FGR fetuses [24]. Although severity-based subgroups were defined, no significant differences in ADC values were found between them. Head circumference was also lower in more severe cases. In our study, UA Doppler PI was significantly higher in FGR compared to SGA fetuses, whereas MCA PI remained similar. Moradi et al. reported no significant UA Doppler PI differences between FGR subgroups, possibly due to differences in study methodology or comparator groups.

Our findings emphasize the potential of MRI, particularly ADC measurements, in distinguishing FGR from SGA and detecting early brain injury. FGR fetuses had higher UA Doppler PI, smaller brain diameters, and lower frontal white matter ADC values. When combined with Doppler studies, MRI may help determine optimal delivery timing and improve outcomes. Although promising, these results need to be validated in larger prospective studies, and standardized protocols are required to support wider clinical application in high-risk pregnancies.

MRI, especially ADC measurement, shows promise in detecting early brain injury in FGR before brain-sparing occurs. Reduced frontal white matter ADC values may indicate early hypoxic effects. Future research should refine MRI protocols, assess long-term neurodevelopmental outcomes, and compare MRI with Doppler to define its added value. Development of standardized guidelines and protocols is essential to support its clinical use, particularly in resource-limited settings. MRI-guided intervention trials may further establish its role in managing high-risk pregnancies.

This study has some limitations. First, the relatively small sample size limited the statistical power and may have reduced the ability to detect small between-group differences, increasing the risk of type II error. Second, the absence of a healthy control group precluded comparison with normative ADC values and limited the interpretation of the clinical significance of the observed findings. Third, the retrospective design may have introduced selection bias. Another limitation of this study is the lack of formal inter-observer reliability analysis, since all measurements were performed by a single observer. Although intra-observer measurements were repeated and carefully standardized using predefined neuroanatomical landmarks and consistent ROI sizes, formal reliability coefficients were not calculated. Therefore, some degree of operator-dependent variability cannot be entirely ruled out. Another important limitation of this study is the lack of long-term neurodevelopmental follow-up. Although neonatal outcomes such as Apgar scores and NICU admission provide useful early clinical information, they cannot reflect long-term developmental trajectories. Therefore, the clinical significance of MRI-detected brain changes cannot be fully determined based on the present data. Future prospective studies incorporating standardized neurodevelopmental assessments in childhood are needed to clarify these associations. Nevertheless, our findings provide preliminary evidence that may help guide the design of larger, well-structured prospective studies with standardized measurement protocols and comprehensive long-term follow-up.

## Conclusions

This study demonstrated that fetuses with FGR exhibit smaller biparietal and transverse cerebellar diameters and significantly lower ADC values in the frontal white matter compared to SGA fetuses. These findings suggest that MRI, particularly diffusion-weighted imaging, may play an important role in identifying early brain alterations associated with FGR before the onset of overt clinical signs or Doppler abnormalities. Incorporating MRI into the prenatal assessment of growth-restricted fetuses may aid in optimizing the timing of delivery and improving neonatal outcomes. Further prospective studies are warranted to validate these findings and to explore the long-term neurodevelopmental implications of early MRI-detected brain changes in FGR. 

## Data Availability

The data supporting the findings of this study were derived from retrospective clinical records and contain potentially identifiable patient information. Due to ethical restrictions imposed by local institutional review boards, national data protection regulations, and the risk of patient re-identification, these data cannot be made publicly available. Access to anonymized data may be considered upon reasonable request to the corresponding author, subject to approval by the relevant ethics committees and, where applicable, the establishment of a data sharing agreement.
